# Gene Expression Profiling of the Peritumoral Immune Cell Infiltrate of Penile Squamous Cell Carcinomas

**DOI:** 10.3390/ijms252212142

**Published:** 2024-11-12

**Authors:** Ria Winkelmann, Nina Becker, Regina Leichner, Peter J. Wild, Melanie Demes, Severine Banek, Claudia Döring, Julia Bein

**Affiliations:** 1 Dr. Senckenberg Institutes of Pathology & Human Genetics, Goethe University Frankfurt, Theodor-Stern-Kai 7, 60596 Frankfurt am Main, Germany; nina.becker@unimedizin-ffm.de (N.B.); regina.leichner@unimedizin-ffm.de (R.L.); peter.wild@unimedizin-ffm.de (P.J.W.); melanie.demes@unimedizin-ffm.de (M.D.); claudia.doering@ukffm.de (C.D.); julia.bein@ukffm.de (J.B.); 2 Frankfurt Institute for Advanced Studies (FIAS), Ruth-Moufang-Straße 1, 60438 Frankfurt am Main, Germany; 3 Department of Urology, Goethe University Frankfurt, Theodor-Stern-Kai 7, 60596 Frankfurt am Main, Germany; severine.banek@unimedizin-ffm.de

**Keywords:** penile squamous cell carcinomas, NanoString, HPV, immunohistochemistry, gene expression, immune cell infiltrate

## Abstract

Penile carcinomas are rare tumors in Europe and need further investigations due to their inferior prognosis in late tumor stages. The presence of disparate immune cell infiltrates was observed in these tumors, which were subsequently demonstrated to give rise to divergent tumor prognoses. The objective was to further characterize this immune cell infiltrate with the use of immunohistochemistry and RNA expression. A total of twelve well-characterized cases of penile squamous cell carcinomas with known infection status by human papillomavirus (HPV) and p16 status were assessed. The cases were classified according to their morphological characteristics, including those exhibiting a pronounced peritumoral immune cell infiltrate and those with less peritumoral immune cell infiltration. The generation of RNA expression data was conducted using the nCounter^®^ PanCancer Immune Profiling Panel. Computational models were employed to calculate the proportions of immune cells. To corroborate the findings, an immunohistochemical analysis was conducted using antibodies against CD20, CD3, CD4, CD8, MUM1, CD68, and CD117. Our cases were clustered according to the immune cell infiltrate detected via histology in a group with less immune cell infiltrate density and in a group with increased immune cell infiltrate density. Generally, all immune cells showed an increased amount in the group with pronounced immune cell infiltrate density. The clusters were found to relate to cell functions, the complement system, cytotoxicity, pathogen defense, regulation, and T-cell functions. In cases exhibiting a pronounced immune cell infiltrate, the top three genes that exhibited the greatest upregulation were *GZMA*, *MICB*, and *GNLY*. No relationship to HPV infection status was demonstrated. Immunohistochemistry validated the data gained via RNA expression and showed a correlation with EPIC and Cibersort. The clustering of cases based on immune cell infiltrate density revealed significant distinctions between groups with lower and higher immune cell infiltrate density. The group with increased immune cel infiltrate density showed a greater abundance of immune cells, aligning with key functions like cytotoxicity, pathogen defense, and T-cell regulation. Among these cases, the genes *GZMA*, *MICB*, and *GNLY* were significantly upregulated, suggesting their involvement in an increased immune response. The role of HPV infection status in our cases with regard to the peritumoral immune cell infiltrate remains inconclusive.

## 1. Introduction

Penile carcinomas are rare tumors in Europe. Therefore, due to limited tissue availability, data are sparse on this humiliating disease. In most cases, penile carcinomas are squamous cell carcinomas, which can show an association with infection by human papillomavirus (HPV). Assuming that the infection status would induce an immune reaction of the host against the tumor, we studied the immune cell infiltrate of penile carcinomas on a group of patients condensed on a tissue micro array (TMA) with the use of hematoxylin and eosin (HE) staining and immunohistochemistry. We discovered that there is a difference in the immune cell infiltrate between cancer and non-cancer tissue and that a decreased immune cell infiltrate is associated with worse overall survival in our cohort of penile carcinomas in Frankfurt am Main, Germany [1,2]. We also analyzed the HRD score in penile carcinoma cell lines [3]. Due to the impaired quality of nucleic acids, the analysis of our retrospective cohort was difficult, and we experienced drop outs. Therefore, in order to gain deeper insights into penile carcinomas and their tumor characteristics with regard to gene expression, we chose NanoString as a robust technique for our cohort, aiming to gain deeper insights into the peritumoral immune cell infiltrate on a well-characterized cohort.

## 2. Results

### 2.1. p16 Immunohistochemistry and HPV Status

As previously described, we depicted the immune cell infiltrate density in our cohort. Four of the twelve cases exhibited a moderate-to-high density of intratumoral immune cells, as indicated by a score of 2 or 3 on the aforementioned scale. The remaining eight cases exhibited a minimal infiltration of immune cells with a score of 1.

The p16 immunohistochemistry demonstrated a block-like positivity in five of the twelve cases, indicating a potential infection by a high-risk HPV strain. Two of the cases with a prominent immune cell infiltrate exhibited p16 positivity, while three cases with a low immune cell infiltrate also demonstrated p16 positivity.

The HPV status, determined via the VisionArray^®^ HPV Chip 1.0 system, indicated that four out of the twelve cases exhibited evidence of HPV infection. Two cases demonstrated infection by HPV16, exhibiting block-like p16 positivity. One case exhibited dual infection by HPV16 and 56, which also demonstrated p16 positivity. Conversely, one case with HPV infection by HPV68 exhibited p16 negativity. Additionally, two cases demonstrated p16 positivity but lacked a confirmed HPV infection via the VisionArray^®^ HPV Chip 1.0 system. The aforementioned results are summarized in Supplementary Table S1 (Supplementary Table S1: Peritumoral immune cell infiltrate density, p16 status and HPV infection status via VisionArray^®^ HPV Chip 1.0).

### 2.2. Results of the NanoString nCounter^®^ Analysis

To eliminate degradation artifacts due to long storage times, the most recent cases of penile carcinomas were selected from our archive. In a total of twelve investigated cases, eleven cases yielded good-quality data, while one case was excluded due to inferior expression results. The HE-stained sections of all investigated cases are presented in Supplementary Figure S1.

**Figure 1:**
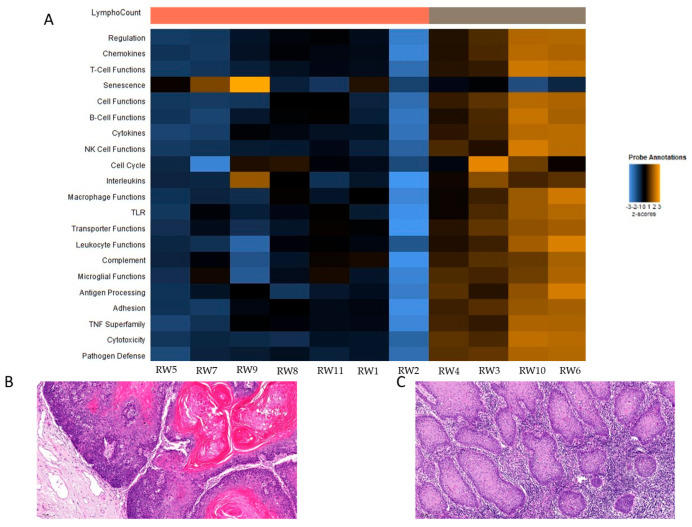
(**A**) The depicted heatmap is an overall cluster analysis summarizing NanoStrings’ gene sets included in the applied panel. A detailed visualization of the gene sets are demonstrated in Supplementary Figure S2 (Supplementary Figure S2: A Heatmap of normalized data for genes involved in cell functions (A), the complement system (B), cytotoxicity (C), pathogen defense (D), regulation (E), and T-cell functions (F). Orange indicates high expression and blue indicates low expression). The heatmap shows pathway scores. This plot is an overview of how the pathway scores change across samples. Orange indicates high scores; blue indicates low scores. Scores are displayed on the same scale via a z-transformation. The values are normalized log2 count data. Every column represents one sample. The sample name, which is also noted in Supplementary Figure S1 (Supplementary Figure S1: HE stained sections of all cases investigated representing low and high lymphocyte count. Magnification 10 fold), is presented beneath the columns. A cluster can be noted according to the immune cell infiltrate density in cases with less immune cells. (**B**) HE staining, magnification 10-fold; case: RW2y exemplary and in more immune cells; (**C**) HE staining, magnification 10-fold; RW10 exemplary. The name LymphoCount refers to the immune cell infiltrate density and shows a separation of the cases with a lower number of immune cells (cases RW5, RW7, RW9, RW8, RW11, RW1) and with an abundance of immune cells (cases RW4, RW3, RW10, RW6).

**Figure 2:**
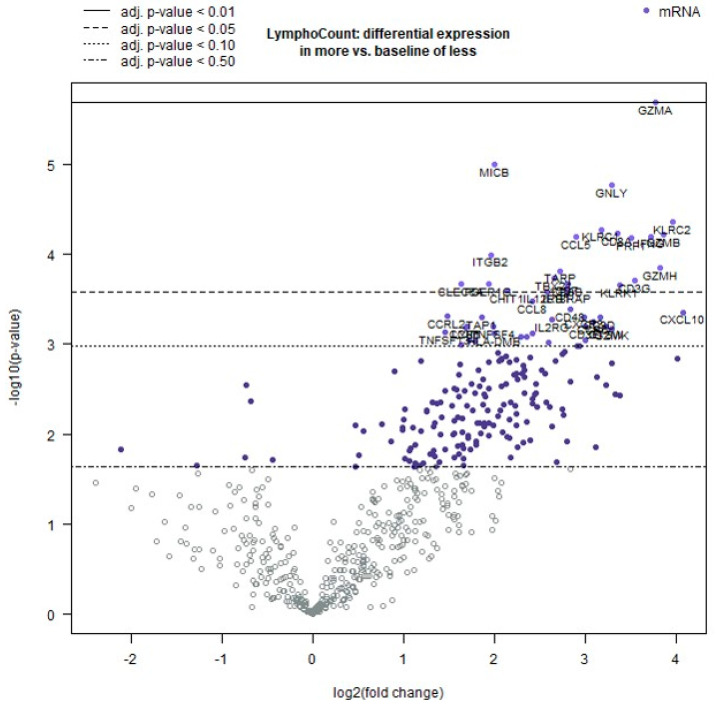
The volcano plot depicts the −log10 (*p*-value) and log2-fold change in each gene in relation to the selected covariate. Genes with high statistical significance are located at the upper portion of the plot, above the horizontal lines, while genes with high differential expression are situated on either side. The horizontal lines represent various false discovery rates (FDRs) or *p*-value thresholds depending on whether the *p*-values have been adjusted. Genes are colored if the resulting *p*-value is below the specified FDR or *p*-value threshold. The 40 most statistically significant genes are labeled in the plot. The plot demonstrates the differential expression between cases with less immune cell infiltrate and with pronounced immune cell infiltrate, as depicted by “LymphoCount”.

The remaining eleven cases were grouped according to the presence and density of peritumoral immune cells. Four cases (4/11) exhibited a higher immune cell infiltrate (immune cell infiltrate score 2 and 3), while seven cases (7/11) demonstrated a lower density of immune cells in the peritumoral region (immune cell infiltrate score 1), as shown in Figure 1 and Figure 2 depicted as “LymphoCount”. In the group with a pronounced immune cell infiltrate, a tumor relapse was observed in one case (1/4). In contrast, in the group with a less pronounced immune cell infiltrate, a relapse was noted in three cases (3/7), indicating a tendency towards a higher incidence of relapses in the group with decreased immune cell infiltrate. The data on the genetic background of the patients and their prior tumor history were not available for all patients included in the study. It is possible that this lack of information may have affected the results regarding the density of immune cell infiltrates.

A supervised hierarchical clustering analysis was conducted to examine the relationships between the cases based on their immune cell infiltrate density. The heatmaps were constructed from the normalized data using a scale that ensures that all genes have an equal variance. The resulting clusters were found to relate to cell functions, the complement system, cytotoxicity, pathogen defense, regulation, and T-cell functions. The results are illustrated in Figure 1 and in more detail in Supplementary Figure S2. Tumors investigated with a higher density of immune cell infiltrate exhibited elevated gene expression levels for the aforementioned functions.

The most significantly differentially expressed genes are presented in Figure 2 using a volcano plot. Among these, *GZMA*, *MICB*, and *GNLY* are the top three genes mentioned. 

All results are presented in Supplementary Table S2 (Supplementary Table S2: Detailed results for RNA Seq Data representing the log2 fold change, p-value, corrected p value, gene set istrubution and probe I.D.). The *p*-values were corrected via the false discovery rate method by Benjamini–Yekutieli. Detailed expression data with corrected *p*-values are presented in Supplementary Table S2. In summary, 258 genes showed differential expression levels, and the corrected *p*-value showed 23 genes, with *p* > 0.05.

To estimate the immune cell infiltrate proportions in our probes, we applied EPIC [4], Cibersort [5], and quanTIseq [6] in our tumor probes.

**Figure 3:**
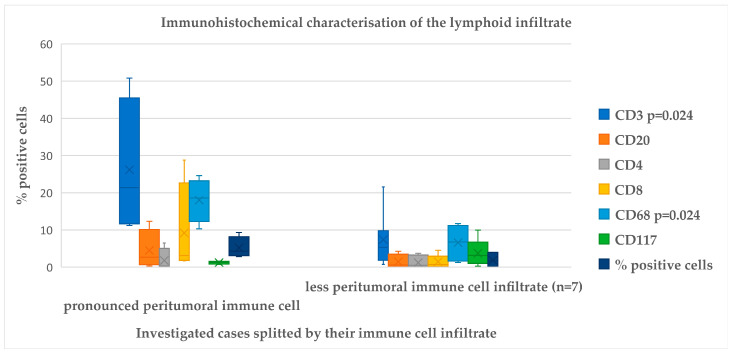
Immunohistochemical characterization of the lymphoid infiltrate. The diagram shows the difference in the immune cell composition as depicted by immunohistochemistry with regard to the amount of immune cell infiltrate in the investigated cases.

Furthermore, we employed immunohistochemistry to our probes with antibodies, which are routinely applied in our laboratory to describe the peritumoral immune cell infiltrate at the expression level as a proof of concept. The composition of the peritumoral immune cell infiltrate is presented in Figure 3.

The expression levels of CD3 and CD68 were found to be significantly different between the two groups (pronounced peritumoral immune cell infiltrate n = 4 and less peritumoral immune cell infiltrate *n* = 7) according to immunohistochemistry with the antibodies applied, as determined by the Mann–Whitney U test (*p* = 0.024 for both markers). The effect strength for both markers is 0.5 according to the criteria established by Cohen [5]. The percentage of positive cells was correlated with the percentage of positive cells obtained via computational methods. Cibersort provides the most comprehensive list of cells and their distribution in the peritumoral immune cell infiltrate. A notable correlation was observed between Cibersort and immunohistochemistry for CD3, CD8, and CD117. Similarly, a significant correlation was demonstrated between EPIC and immunohistochemistry for CD20, CD3, and CD8. However, the only significant correlation was identified for CD3 when using quanTIseq. The correlation between immunohistochemistry and the calculated immune cell infiltrate is presented in Table 1.

## 3. Discussion

The tumor microenvironment is a subject of study in a multitude of tumors, and it is subject to a variety of influences, including the status of infection by HPV. It is established that penile carcinomas can be associated with infection by HPV [6]. By conventional HE staining, the assessed immune cell infiltrate density in penile carcinomas was shown to have a significant association with survival in our previous study [1]. Therefore, further examination of the immune cell infiltrate on a molecular level was aimed for by us. We studied twelve well-characterized cases of penile squamous cell carcinomas out of our archive, applying the RNA expression analysis using the nCounter^®^ PanCancer Immune Profiling Panel.

The NanoString technique is employed for gene expression profiling on FFPE tissue samples, with a direct quantification of target molecules. The procedure does not necessitate the inclusion of a standard, nor does it require any amplification steps or replicates. Furthermore, the construction of a library is not required and no enzymatic steps are involved [7].

As the NanoString technique produces robust results on FFPE material, we experienced one case dropping out, maybe due to fixation or storage [8].

In total, eleven cases were evaluable. The cases were then divided into two distinct groups, one comprising those with a minimal peritumoral immune cell infiltrate and the other with a pronounced immune cell infiltrate. The data given here are limited due to the small sample size.

In two cases with a pronounced immune cell infiltrate, p16 positivity was observed, suggesting the possibility of infection with high-risk HPV. It is hypothesized that an HPV infection may serve as a trigger for an increased immune cell infiltrate in penile cancer. Additionally, tumors are known to have a differing immune cell infiltrate, and CD8-positive T-cells are suspected to play a pivotal role in this complex mechanism [9]. However, additional testing with the VisionArray^®^ HPV Chip did not confirm this assumption. Instead, the cases were determined to be negative for high-risk HPV infection. It can be reasonably inferred that the immune cell infiltrate was potentially elicited by the tumor itself in these cases.

Our findings revealed that tumors with pronounced immune cell infiltrate had an increased expression of markers related to cell functions, the complement system, cytotoxicity, pathogen defense, regulation, and T-cell functions.

Therefore, we suspect an increased activity of human killer cells that exhibit cyctotoxic granules containing perforins and granulysin, as well as granzymes [10].

Among the genes that were upregulated, granzyme A and B were listed early. The expression of granzyme was described in penile cancer and was correlated with an increased number of tumor-infiltrating lymphocytes [11]. Furthermore, elevated levels of granzyme B were linked to more favorable outcomes in individuals diagnosed with penile squamous cell carcinoma according to the literature [12]. *GZMA* is a component of the interferon gamma (*IFN-γ*) signature, which has been demonstrated to serve as a predictor of response or non-response to anti-PD-1 therapy as part of an immune-related gene expression signature in ten distinct tumor types [13]. Interferon gamma (*IFN-γ*) is produced by activated T-cells or natural killer T-cells, which in turn activate downstream molecules in dendritic cells and macrophages. This facilitates the recruitment of additional CD8+ T-cells. It has been demonstrated that *IFN-γ* induces the upregulation of major histocompatibility complex (*MICB*, *MHC*) molecules and other components of the immunoproteosome [13]. In penile cancer, the loss of human leukocyte antigen (*HLA*) expression has been demonstrated to function as a mechanism of immune evasion and has been identified as a predictor of poor survival in patients with human papilloma virus (HPV) negativity [14]. In other cancers, such as colorectal cancer, an association between *MICB* expression and a favorable prognosis has been documented [15]. Moreover, interferon gamma has been demonstrated to upregulate a multitude of checkpoint inhibitors, including programmed death ligands 1 and 2, on the surface of macrophages, dendritic cells, and tumor cells [13].

In our cohort, we observed a tendency towards an increased amount of tumor relapse in the group of patients with a less pronounced immune cell infiltrate, which serves to enhance the significance of the immune system in the tumor setting. Understanding the immune cell infiltrate in neoplasias is a key factor to generate immune-based therapies. The marker PD-L1 has been demonstrated to have prognostic utility in patients with penile cancer, irrespective of HPV status. This suggests a potential role for PD-L1 in therapeutic settings, although this is not yet a routine application. Nevertheless, the application of immune therapies could also prove beneficial in earlier disease stages, which should be considered in future investigations involving the immune landscape in primary diagnosis [16].

Moreover, it has been proposed that HPV may contribute to the peritumoral immune cell infiltrate. It is possible that this was not demonstrated in our investigation due to the limited sample size. Nevertheless, the implementation of HPV vaccination may prompt another component of the intricate immune system to elicit an antitumor response [16].

Furthermore, the results of our investigation support the potential use of immune-based therapies, such as adoptive T-cell therapies or a combination of therapies, to enhance the efficacy of immune-based therapies driven by the activity of cytotoxic T lymphocytes.

It is important to note, however, that there is currently no evidence to suggest whether the upregulated genes originate from the tumor itself or the immune cell infiltrate.

The assessment of immune cell infiltration with computational methods is a process that can be performed by software algorithms, as reviewed in [17]. There are several tools that can be applied, such as the followng:

QuanTIseq generates scores that can be interpreted as a cell-type fraction, relative to all cells in a sample [18]. With TIMER, tumor-infiltrating immune cell estimates are not normalized to sum up to one. Consequently, they cannot be interpreted directly as cell fractions or compared across different immune cell types and data sets. However, they can be considered as arbitrary units that are comparable between samples, irrespective of the cancer type [19]. Cibersort simultaneously estimates the proportion of 22 different cell types and phenotypes. It also assesses the relative abundance of immune cells in relation to the total immune cell content. The Cibersort absolute mode provides an arbitrary unit score that reflects the absolute proportion of each cell type [20]. The results of the EPIC estimates demonstrated a high degree of concordance with the cell fractions obtained through flow cytometry, both for the immune cells and the uncharacterized cells. The cell fractions were calculated relative to the total number of cells in the sample [4]. The MCP counter provides scores in arbitrary units that are only comparable between samples, but not between cell types [21]. The tools chosen allowed both inter- and intra-sample comparisons. Additionally, the methodologies described are evaluated against RNAseq data and array data. Given that a reduced panel of genes was applied to the tumors tested, a correlation with immunohistochemistry was established. The EPIC and Cibersort method demonstrated the greatest degree of comparability with the ground truth, as depicted by immunohistochemistry. Nevertheless, Cibersort was selected as the deconvolution method, which had been previously described in the literature using this panel before in a study that investigated ten different cancer types [7]. Our findings demonstrate that there is at least one additional deconvolution method that can be reasonably applied in this setting. The composition of the immune cell infiltrate mirrored the histology.

There is another study investigating advanced penile carcinomas using the NanoString technique. The findings thus far indicate that the genes *MAML2*, *KITLG*, and *JAK1* are associated with poor overall survival, while the gene *FANCA* is associated with better overall survival [22]. In this investigation, a distinct gene expression panel was employed on the tumor specimen, which did not solely encompass advanced tumor sizes on our cohort. Moreover, the immune cell infiltrate was a focal point of investigation by our group. Of the genes of interest highlighted by Necchi et al., it was determined that KIT and JAK1 are subject to differential regulation within our cohort (see Supplementary Figure S3 Heatmap demonstrating the regulation of the genes KIT and JAK1 in our cohort of 11/12 penile squamous cell carcinomas). However, the limited sample size of our cohort precludes the ability to ascertain the impact of these differential regulatory events.

A limitation of this study is the limited sample size.

## 4. Materials and Methods

### 4.1. Patient Collective

We retrospectively screened the archive of the Dr. Senckenberg Institute of Pathology, Frankfurt am Main, for paraffin block material (FFPE) from surgical cases of penile squamous cell carcinomas. Twelve cases with sufficient FFPE material were retrieved from the biobank of the Dr. Senckenberg Institute of Pathology after a pathological review of newly obtained HE whole-slide images presenting the current biomaterial histology. The tumor classification was performed according to the WHO classification system, fifth edition [23], with the clinicopathological characteristics shown in Table 2. The p16 status of the tumors was determined from the initial pathology report. Furthermore, the peritumoral immune cell infiltrate was evaluated visually using HE staining for all the probes analyzed, as previously described [1] as follows: few immune cells—immune cell infiltrate score 1; medium amount of immune cells—immune cell infiltrate score 2; and many immune cells—immune cell infiltrate score 3. The word LymphoCount is used for the immune cell infiltrate score in Figure 1 and Figure 2. The median overall survival, as defined by the date of initial diagnosis to last follow up, was 18 months (3–64 months). One patient died of unknown reasons. Additional chemotherapy was administered to three patients who received lymphadenectomy.

### 4.2. Ethical Statement

FFPE material from resection specimens and biopsy probes was processed following diagnostic procedures. The tissue and pseudonymized patient data used in this study were provided by the University Cancer Centre Frankfurt (UCT). Written informed consent was obtained from all patients, and the retrospective study was approved by the Ethics Committee of the Medical Faculty of the University of Frankfurt (Ethikkommission des Fachbereichs Medizin der Goethe-Universität) in accordance with the Declaration of Helsinki in its current version (project number: SUG-1-2024; date: 4 March 2024).

Nucleic acid extraction and quality control RNA was extracted from tumor punches taken subsequent to a pathological review of the current HE-stained slides. RNA was extracted using the Covaris truXTRAC FFPE total NA kit (Column # 520220, Covaris, MA, USA) in accordance with the manufacturer’s instructions. The concentration and degradation of the RNA was evaluated using the Agilent 2100 bioanalyzer (Agilent, Santa Clara, CA, USA).

The DNA was extracted and purified from FFPE samples using Promega’s Maxwell^®^ RSC DNA FFPE Kit (Promega, Madison, USA) in accordance with the manufacturer’s instructions. The DNA concentration was determined using the Qubit 4.0 fluorometer with the Qubit 1X dsDNA HS Assay Kit (Life Technologies, Eugene, OR, USA). The DNA was subjected to screening for degradation using the Agilent TapeStation 4150 (Agilent, Santa Clara, CA, USA). The DIN values range from 1 to 10, with 10 representing completely intact DNA and 1 representing completely degraded DNA.

### 4.3. Detection of the HPV Infection Status

The VisionArray^®^ HPV Chip 1.0 system (ZytoVision GmbH, Bremerhaven, Germany) was utilized for the detection of the HPV infection status in accordance with the instructions provided by the manufacturer. The VisionArray^®^ HPV Chip detects DNA from 41 clinically relevant HPV genotypes, namely 12 high-risk (HPV16, 18, 31, 33, 35, 39, 45, 51, 52, 56, 58, and 59), 12 probably high-risk (HPV26, 34, 53, 66, 67, 68a, 68b, 69, 70, 73, 82IS39, and 82MM4), and 17 low-risk (HPV6, 11, 40, 42, 43, 44, 54, 55, 57, 61, 62, 72, 81CP8304, 83MM7, 84MM8, 90 and 91) genotypes. In accordance with the manufacturer’s instructions, the VisionArray HPV PreCise Master Mix (ZytoVision #ES-0007) was utilized, followed by the detection kit (ZytoVision, #VK-0003). For the evaluation of the chip, the VisionArray MultiScan E4302 software with a threshold of 25 was employed.

### 4.4. NanoString nCounter^®^ Analysis

We applied the nCounter^®^ PanCancer Immune Profiling Panel (NanoString Technologies, Inc., Seattle, WA, USA) to our probes according to the manufacturer’s instructions. Data analysis was carried out using nSolver™ Analysis Software (https://nanostring.com/products/ncounter-analysis-system/ncounter-analysis-solutions/ nSolver 4.0 for Windows 32-bit (accessed on 1 May 2024)) and the advanced Analysis Module (https://nanostring.com/products/ncounter-analysis-system/nsolver-advanced-analysis-software/ (accessed on 1 May 2024)). The expression of 770 genes was analyzed.

The background correction with the negative controls was set to 20 and the normalization with the positive controls was set to standard parameters. In order to gain insights into the proportions of immune cells in our tissue, we evaluated the utility of several computational tools designed to calculate these proportions. The ground truth for these calculations is RNASeq data. However, the data used here are limited and therefore, we chose to calculate immune cell proportions using EPIC [4], Cibersort [20], and quanTIseq [5] on our cohort because both inter- and intra-sample comparisons are possible with these models.

### 4.5. Immunohistochemical Analysis

Furthermore, we applied the antibodies CD20, clone L26 (Agilent, Dako, Santa Clara, CA, USA); CD3, polyclonal (Agilent, Dako, Santa Clara, CA, USA); CD4, clone 4B12 (Agilent, Dako, Santa Clara, CA, USA); CD8, clone C8/144B (Agilent, Dako, Santa Clara, CA, USA); MUM1, clone MUM1p (Agilent, Dako, Santa Clara, CA, USA); and CD68, clone KP1 (Agilent, Dako, Santa Clara, CA, USA). All of the above-mentioned antibodies were applied as ready-to-use dilutions according to the manufacturers’ instructions. CD117, clone c-Kit polyclonal (Agilent, Dako, Santa Clara, CA, USA) was diluted at 1:200. The applied stainer was the DAKO Autostainer Link 48 (Agilent, Santa Clara, CA, USA). The antibodies mentioned were routinely applied and validated in the authors’ laboratory. The slides were digitized using a 20× magnification on the brightfield slide scanner (Pannoramic Scan II, 3D Histech, Budapest, Hungary). The digitized slides were then imported into QuPath 0.5.0 [24]. The spots were marked, and cells were detected using the fast cell count option with preset variables. The results were then subjected to a visual examination by a pathologist.

### 4.6. Statistical Analysis

We applied Pearson’s correlation, two-sided t-tests and the Mann–Whitney U test on our probes using IBM^®^ SPSS^®^ Statistics 26 (IBM Corp., IBM SPSS Statistics for Windows, Armonk, NY, USA). Statistical significance was considered at *p* < 0.05.

## 5. Conclusions

Gene expression data mirror immune cell infiltrate density in our cohort of penile squamous cell carcinomas and show a distinct immune cell composition in our cases associated with key functions like cytotoxicity, pathogen defense, and T-cell regulation. Notably, the upregulation of genes such as *GZMA*, *MICB*, and *GNLY* was prominent in this group, indicating potential biomarkers for immune activity.

## Figures and Tables

**Table 1:** Correlation between immunohistochemical staining and computational models.

Computational Method	Immunohistochemistry	Pearson-Korrelation	sig. (2-Sided)
EPIC	CD20	0.697	**0.017**
	CD3	0.815	**0.002**
	CD4	0.337	0.311
	CD8	0.623	**0.040**
	CD68	0.487	0.129
Cibersort	CD20	0.179	0.599
	CD3	0.672	**0.024**
	CD4	0.120	0.725
	CD8	0.679	**0.021**
	CD68	−0.128	0.708
	CD117	0.636	**0.036**
	MUM1	−0.042	0.903
quanTIseq	CD20	−0.074	0.828
	CD3	0.852	**0.001**
	CD4	NA	
	CD8	−0.189	0.578
	CD68	−0.147	0.666

NA: Not analysable.

**Table 2:** Clinicopathological characteristics of invasive penile carcinomas (*n* = 12).

Clinicopathological Characteristics of Invasive Penile Carcinomas	Categorization	N	%
Age (range 39–88 years)	≤74 years	6	50
	>74 years	6	50
Pathological assessed tumor stage (pT)	pT1a	3	25
	pT1b	2	17
	pT2	4	33
	pT3	3	25
	pT4	0	0
Pathological assessed nodal status (pN)	pN0	6	50
	pN > 0	3	25
	pNX	3	25
Grading (G)	G1	2	17
	G2	6	50
	G3	4	33
Lymphovascular invasion (L)	L0	7	58
	L1	5	42
Vascular invasion (V)	V0	12	100
	V1	0	0
Perineural invasion (Pn1)	Pn0	11	92
	Pn1	1	8
Resection status (R)	R0	11	92
	R1	0	0
	R1 (is)	1	8
Infiltration pattern	infiltrative pattern	2	17
	pushing margin	10	83
Morphological tumor type	usual	11	92
	basaloid	1	8
Immunohistochemistry (IHC)			
p16 IHC	negative	7	58
	positive	5	42

## Data Availability

The datasets used and/or analyzed during the current study are published in the Supplementary Materials.

## Supplementary Materials

The following supporting information can be downloaded at: https://www.mdpi.com/article/10.3390/ijms252212142/s1.

## References

[1] Müller T., Demes M., Lehn A., Köllermann J., Vallo S., Wild P.J., Winkelmann R. (2021). The peri- and intratumoral immune cell infiltrate and PD-L1 status in invasive squamous cell carcinomas of the penis. Clin. Transl. Oncol..

[2] Hladek L., Bankov K., von der Grün J., Filmann N., Demes M., Vallo S., Wild P.J., Winkelmann R. (2022). Tumor-associated immune cell infiltrate density in penile squamous cell carcinomas. Virchows Arch..

[3] Winkelmann R., Bankov K., Döring C., Cinatl J., Grothe S., Rothweiler F., Michaelis M., Schmitt C., Wild P.J., Demes M. (2022). Increased HRD score in cisplatin resistant penile cancer cells. BMC Cancer.

[4] Racle J., De Jonge K., Baumgaertner P., Speiser D.E., Gfeller D. (2017). Simultaneous enumeration of cancer and immune cell types from bulk tumor gene expression data. ELife.

[5] Cohen J. (1992). Quantitative Methods in Psychology A Power Primer. Psychol. Bull..

[6] Amicuzi U., Grillo M., Stizzo M., Olivetta M., Tammaro S., Napolitano L., Reccia P., De Luca L., Rubinacci A., Della Rosa G. (2024). Exploring the Multifactorial Landscape of Penile Cancer: A Comprehensive Analysis of Risk Factors. Diagnostics.

[7] D’angelo A., Kilili H., Chapman R., Generali D., Tinhofer I., Luminari S., Donati B., Ciarrocchi A., Giannini R., Moretto R. (2023). Immune-related pan-cancer gene expression signatures of patient survival revealed by NanoString-based analyses. PLoS ONE.

[8] Mathieson W., Thomas G. (2019). Using FFPE Tissue in Genomic Analyses: Advantages, Disadvantages and the Role of Biospecimen Science. Curr. Pathobiol. Rep..

[9] Ahmed M.E., Falasiri S., Hajiran A., Chahoud J., Spiess P.E. (2020). The Immune Microenvironment in Penile Cancer and Rationale for Immunotherapy. J. Clin. Med..

[10] Dotiwala F., Lieberman J. (2019). Granulysin: Killer lymphocyte safeguard against microbes. Curr. Opin. Immunol..

[11] Winarti N.W., Arijana I.J.K.N., Tunas I.K., Widyadharma I.P.E. (2021). Tumor Infiltrating Lymphocytes and Expression of Granzyme B in Penile Squamous Cell Carcinoma at Sanglah General Hospital, Bali. Int. J. Med. Rev. Case Rep..

[12] Chu C., Yao K., Lu J., Zhang Y., Chen K., Lu J., Zhang C.Z., Cao Y. (2020). Immunophenotypes Based on the Tumor Immune Microenvironment Allow for Unsupervised Penile Cancer Patient Stratification. Cancers.

[13] Ayers M., Lunceford J., Nebozhyn M., Murphy E., Loboda A., Kaufman D.R., Albright A., Cheng J.D., Kang S.P., Shankaran V. (2017). IFN-γ–related mRNA profile predicts clinical response to PD-1 blockade. J. Clin. Investig..

[14] Djajadiningrat R.S., Horenblas S., Heideman D.A., Sanders J., de Jong J., Jordanova E.S. (2015). Classic and nonclassic HLA class I expression in penile cancer and relation to HPV status and clinical outcome. J. Urol..

[15] Feng Q., Yu S., Mao Y., Ji M., Wei Y., He G., Chang W., Zhu D., Ren L., Xu J. (2020). High MICB expression as a biomarker for good prognosis of colorectal cancer. J. Cancer Res. Clin. Oncol..

[16] Joshi V.B., Spiess P.E., Necchi A., Pettaway C.A., Chahoud J. (2022). Immune-based therapies in penile cancer. Nat. Rev. Urol..

[17] Sturm G., Finotello F., Petitprez F., Zhang J.D., Baumbach J., Fridman W.H., List M., Aneichyk T. (2019). Comprehensive evaluation of transcriptome-based cell-type quantification methods for immuno-oncology. Bioinformatics.

[18] Finotello F., Mayer C., Plattner C., Laschober G., Rieder D., Hackl H., Krogsdam A., Loncova Z., Posch W., Wilflingseder D. (2019). Molecular and pharmacological modulators of the tumor immune contexture revealed by deconvolution of RNA-seq data. Genome Med..

[19] Li T., Fan J., Wang B., Traugh N., Chen Q., Liu J.S., Li B., Liu X.S. (2017). TIMER: A Web Server for Comprehensive Analysis of Tumor-Infiltrating Immune Cells. Cancer Res..

[20] Newman A.M., Liu C.L., Green M.R., Gentles A.J., Feng W., Xu Y., Hoang C.D., Diehn M., Alizadeh A.A. (2015). Robust enumeration of cell subsets from tissue expression profiles. Nat. Methods.

[21] Becht E., Giraldo N.A., Lacroix L., Buttard B., Elarouci N., Petitprez F., Selves J., Laurent-Puig P., Sautès-Fridman C., Fridman W.H. (2016). Estimating the population abundance of tissue-infiltrating immune and stromal cell populations using gene expression. Genome Biol..

[22] Necchi A., Eigl B.J., Yang E.S.-H., Bae S., Chandrashekar D., Chen D., Naik G., Mehta A., Giannatempo P., Colecchia M. (2016). Gene Expression Profiling of Advanced Penile Squamous Cell Carcinoma Receiving Cisplatin-based Chemotherapy Improves Prognostication and Identifies Potential Therapeutic Targets. Eur. Urol. Focus.

[23] International Agency for Research on Cancer (2022). Urinary and Male Genital Tumours.

[24] Bankhead P., Loughrey M.B., Fernández J.A., Dombrowski Y., McArt D.G., Dunne P.D., McQuaid S., Gray R.T., Murray L.J., Coleman H.G. (2017). QuPath: Open source software for digital pathology image analysis. Sci. Rep..

