# Supplementary material for: Gene Expression Profiling of the Peritumoral Immune Cell Infiltrate of Penile Squamous Cell Carcinomas
Source: Int J Mol Sci. 2024 Nov 12;25(22):12142. doi: 10.3390/ijms252212142 (PMC11594387; doi:10.3390/ijms252212142)
Supplement: Supplementary file 1 [file ijms-25-12142-s001.zip › Supplementary Figure S1.pptx]

## Slide 1
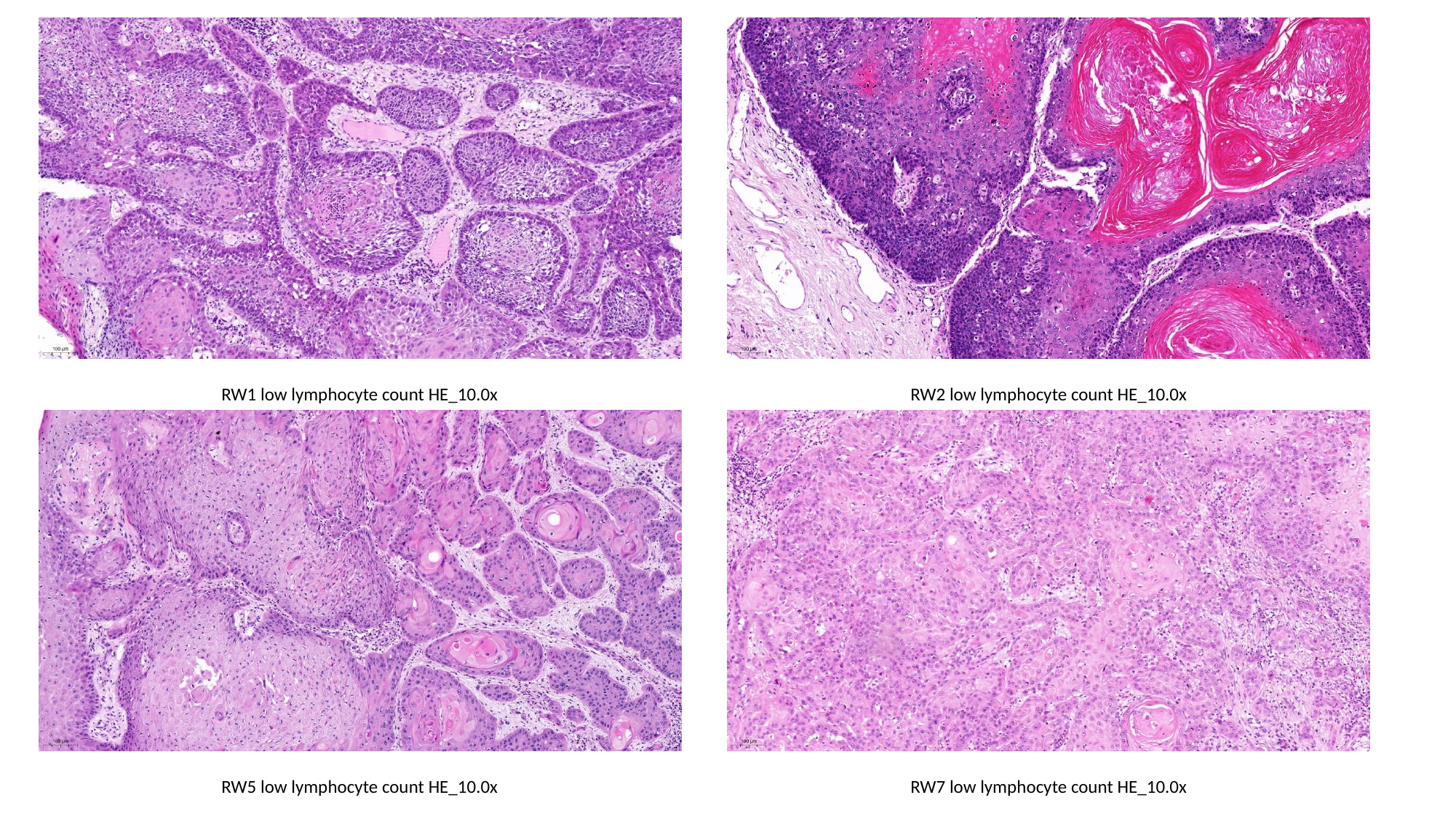

RW1 low lymphocyte count HE_10.0x
RW2 low lymphocyte count HE_10.0x
RW5 low lymphocyte count HE_10.0x
RW7 low lymphocyte count HE_10.0x

## Slide 2
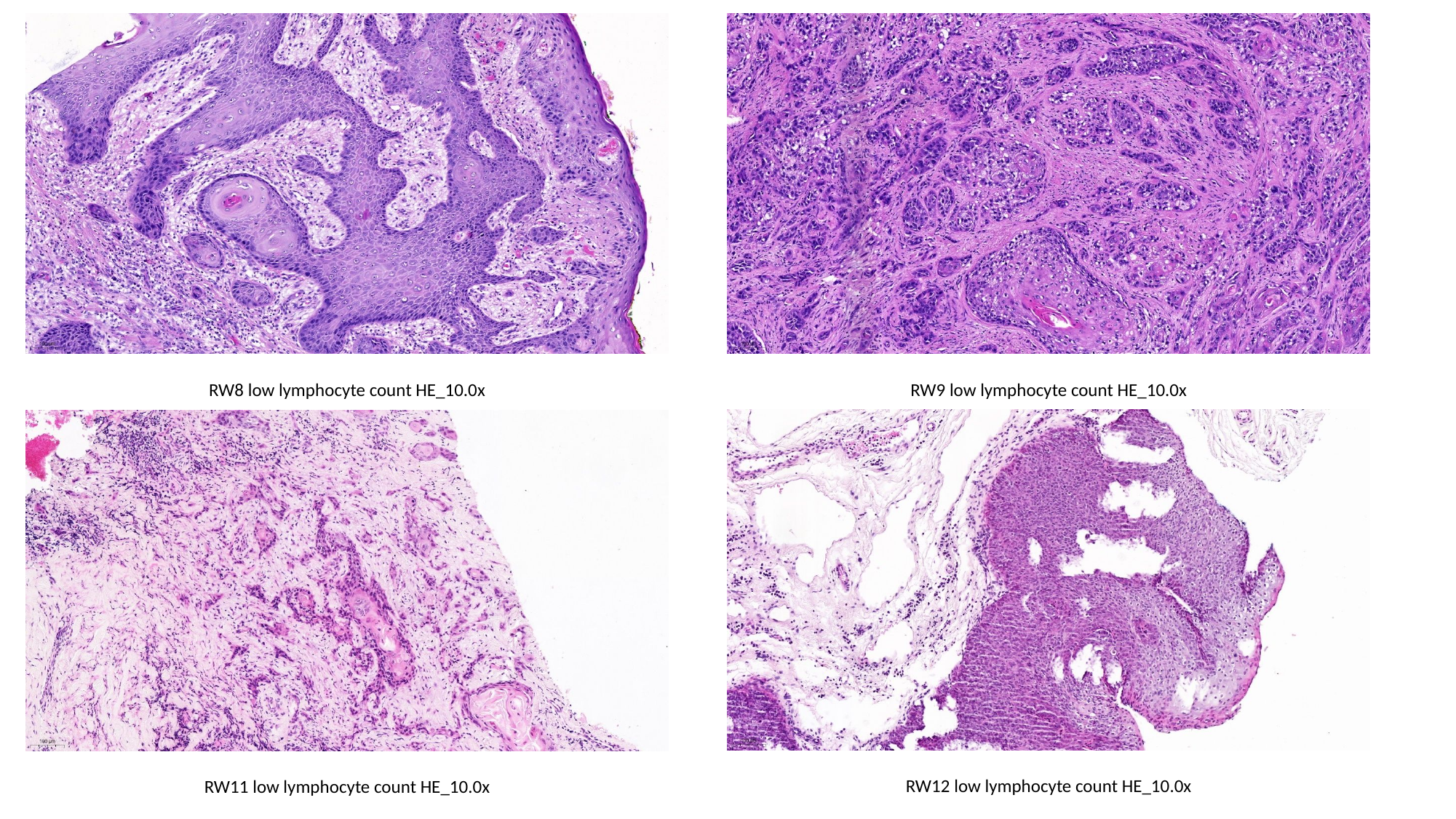

RW8 low lymphocyte count HE_10.0x
RW9 low lymphocyte count HE_10.0x
RW12 low lymphocyte count HE_10.0x
RW11 low lymphocyte count HE_10.0x

## Slide 3
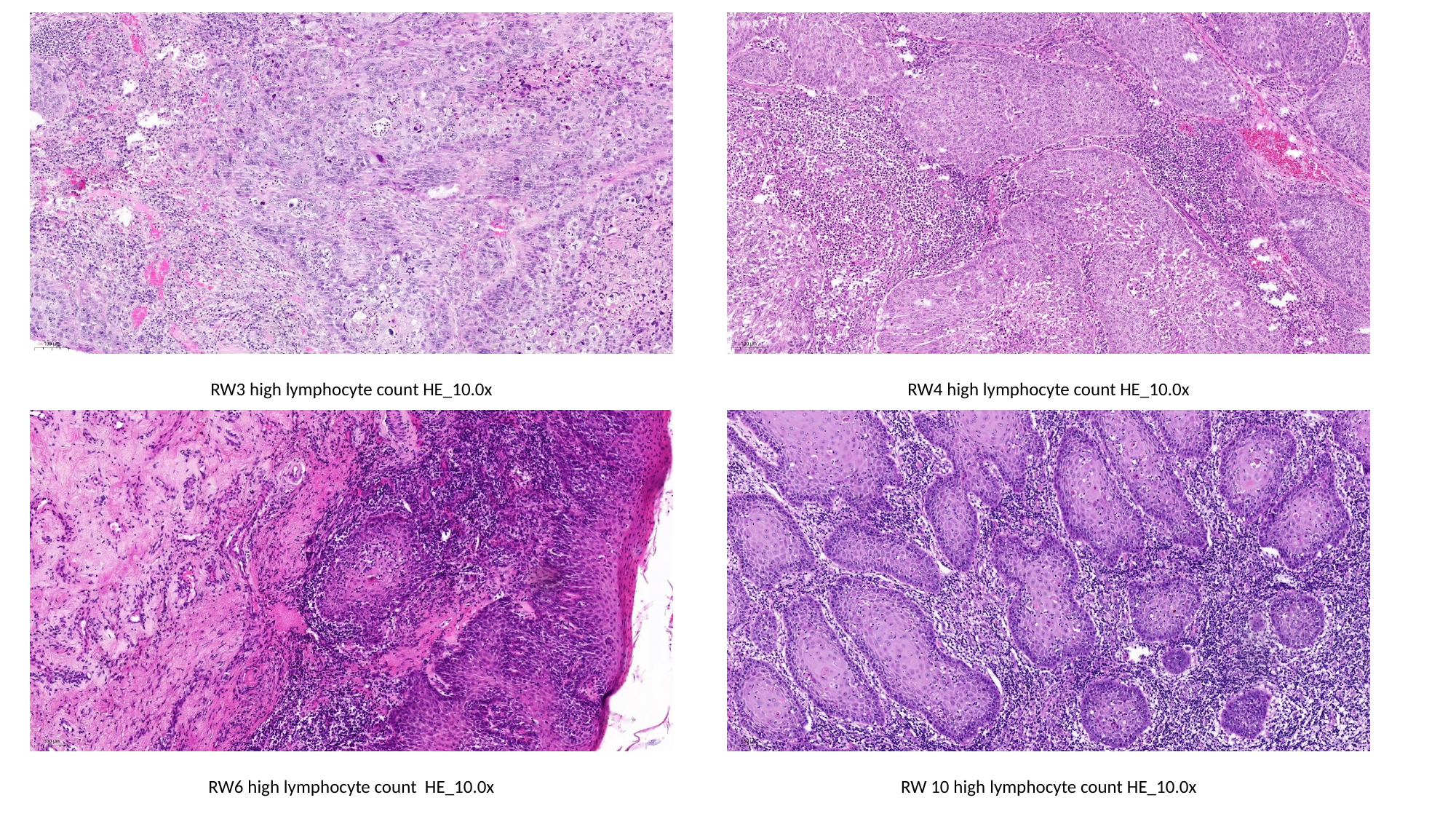

RW3 high lymphocyte count HE_10.0x
RW4 high lymphocyte count HE_10.0x
#
RW6 high lymphocyte count HE_10.0x
RW 10 high lymphocyte count HE_10.0x
