# Supplementary figures and images for: Gene Expression Profiling of the Peritumoral Immune Cell Infiltrate of Penile Squamous Cell Carcinomas
Source: Int J Mol Sci. 2024 Nov 12;25(22):12142. doi: 10.3390/ijms252212142 (PMC11594387; doi:10.3390/ijms252212142)

## Slide 1
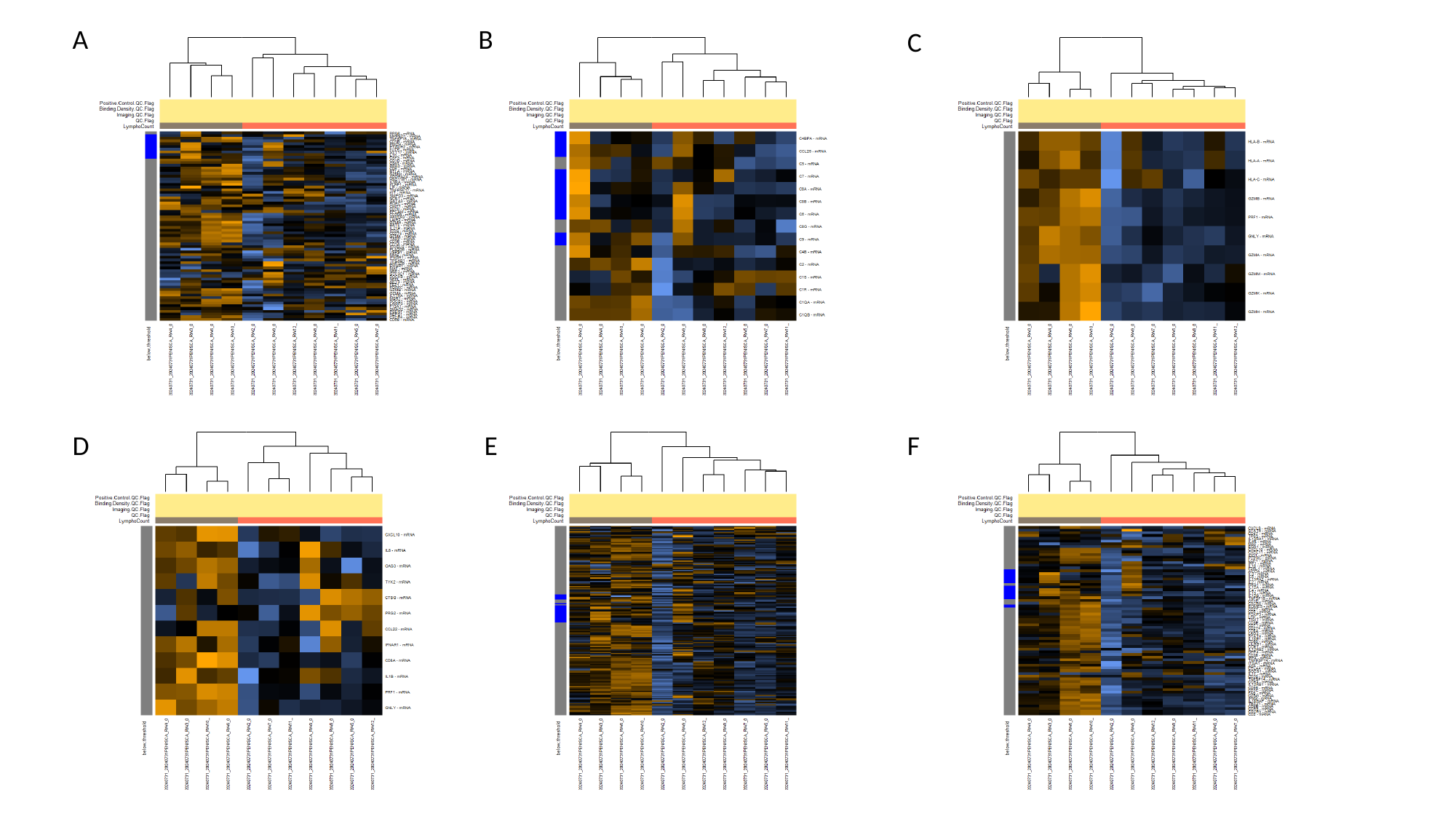

A
B
C
D
E
F

Supplement: Supplementary file 1 [file ijms-25-12142-s001.zip › Supplementary Figure S2 Heatmaps.pptx]

## Slide 1
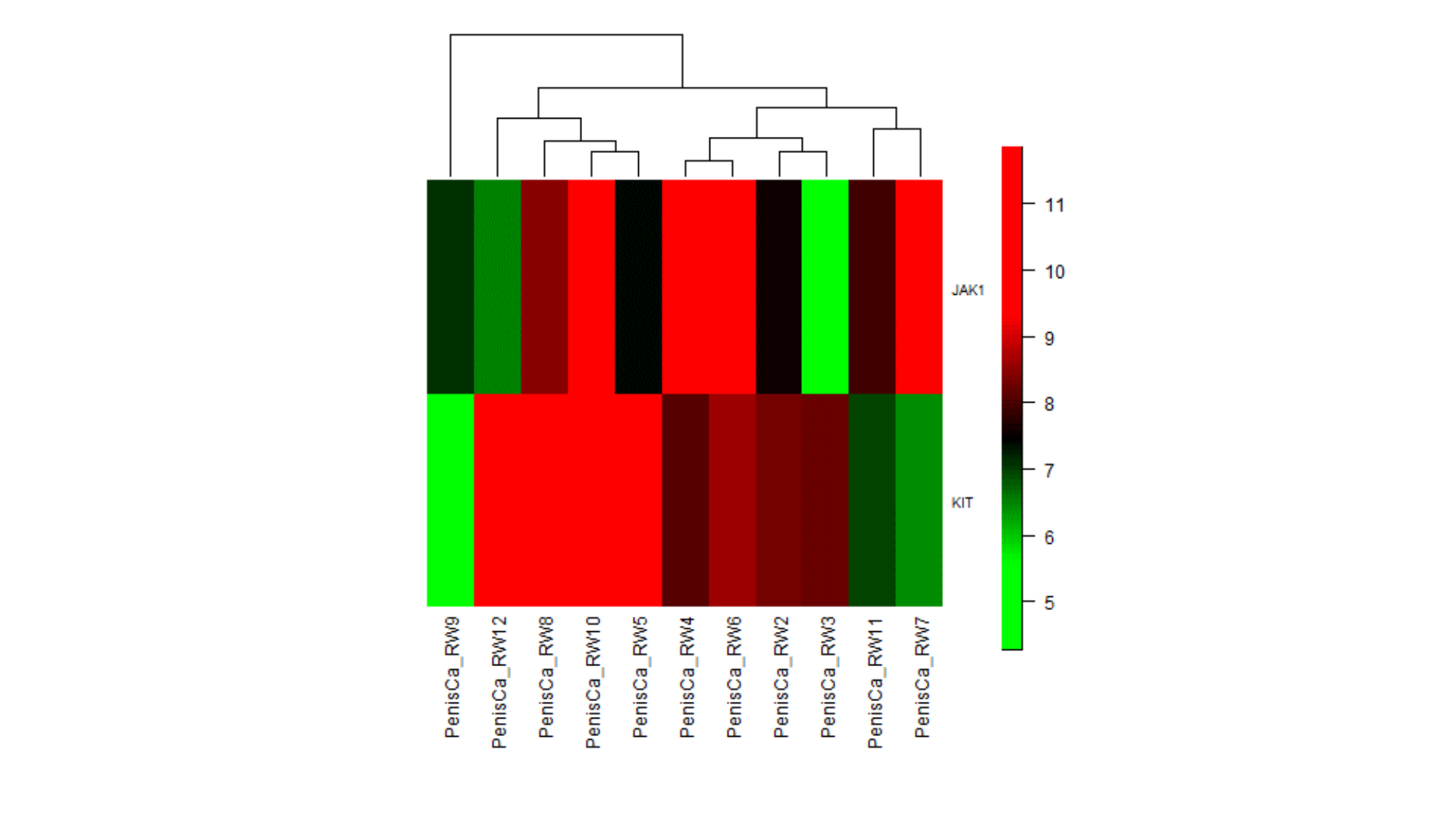

Supplement: Supplementary file 1 [file ijms-25-12142-s001.zip › Supplementary Figure S3.pptx]
